# Centralized and distributed cognitive task processing in the human connectome

**DOI:** 10.1162/netn_a_00072

**Published:** 2019-02-01

**Authors:** Enrico Amico, Alex Arenas, Joaquín Goñi

**Affiliations:** School of Industrial Engineering, Purdue University, West-Lafayette, IN, USA; Purdue Institute for Integrative Neuroscience, Purdue University, West-Lafayette, IN, USA; Departament d’Enginyeria Informàtica i Matemàtiques, Universitat Rovira i Virgili, Tarragona, Spain; School of Industrial Engineering, Purdue University, West-Lafayette, IN, USA; Purdue Institute for Integrative Neuroscience, Purdue University, West-Lafayette, IN, USA; Weldon School of Biomedical Engineering, Purdue University, West-Lafayette, IN, USA

**Keywords:** Brain connectomics, Functional connectivity, Network science, Information theory, Cognitive task processing

## Abstract

A key question in modern neuroscience is how cognitive changes in a human brain can be quantified and captured by functional connectivity (FC). A systematic approach to measure pairwise functional distance at different brain states is lacking. This would provide a straightforward way to quantify differences in cognitive processing across tasks; also, it would help in relating these differences in task-based FCs to the underlying structural network. Here we propose a framework, based on the concept of Jensen-Shannon divergence, to map the task-rest connectivity distance between tasks and resting-state FC. We show how this information theoretical measure allows for quantifying connectivity changes in distributed and centralized processing in functional networks. We study resting state and seven tasks from the Human Connectome Project dataset to obtain the most distant links across tasks. We investigate how these changes are associated with different functional brain networks, and use the proposed measure to infer changes in the information-processing regimes. Furthermore, we show how the FC distance from resting state is shaped by structural connectivity, and to what extent this relationship depends on the task. This framework provides a well-grounded mathematical quantification of connectivity changes associated with cognitive processing in large-scale brain networks.

## Author Summary

A key question in modern neuroscience is how cognitive changes in a human brain can be quantified and captured by functional connectivity (FC). Here we propose a framework, based on Jensen-Shannon divergence, to define “connectivity distance” and to infer about brain network reconfiguration across different tasks with respect to resting state, and to explore changes in centralized and distributed processing in FCs.

Three functional networks (dorsal attention, frontoparietal and DMN) showed major changes in distributed processing and minor changes in centralized processing. Changes in centralized processing depend on the underlying structural connectivity weights and structural path “hiddenness.”

These findings suggest that the cognitive “switch” between resting state and task states is a complex interplay between maximally and minimally distant functional connections, and the underlying structure.

## INTRODUCTION

The progress in neuroimaging methodologies in recent years, together with the rise of publicly available datasets (Van Essen, Smith, et al., 2013; Van Essen, Ugurbil, et al., 2012), has boosted research on quantitative analysis of brain connectivity patterns based on network science. The intuition of modeling the brain as a network (Bassett & Bullmore, 2006; Bullmore & Sporns, 2009; Fornito, Zalesky, & Bullmore, 2016; Sporns, 2011) has rapidly expanded into the scientific area denominated brain connectomics (Fornito, Zalesky, & Breakspear, 2015; Fornito et al., 2016). In brain network models, nodes correspond to gray matter regions (based on brain atlases or parcellations), while links or edges correspond to structural or functional connections. Structural connections are estimated from diffusion-weighted imaging (Bihan, 2003; Tournier, Mori, & Leemans, 2011) data by modeling white matter pathways through tractography algorithms (R. E. Smith, Tournier, Calamante, & Connelly, 2012, 2015; Tournier, Calamante, & Connelly, 2012). Functional connections represent statistical dependencies between brain region time series while subjects are either at rest or performing a task during functional MRI (fMRI) sessions (van den Heuvel & Hulshoff Pol, 2010). These functional associations are usually measured via correlations among fMRI time series to study functional connectivity (FC) in the human brain (Fox & Raichle, 2007; van den Heuvel & Hulshoff Pol, 2010).

These recent advances have led the brain connectivity community to start exploring and quantify differences between resting-state FCs and task-based FCs (Cole, Bassett, Power, Braver, & Petersen, 2014; Cole et al., 2013; Cole & Schneider, 2007; Gonzalez-Castillo et al., 2012; Gratton, Laumann, Gordon, Adeyemo, & Petersen, 2016; Krienen, Yeo, & Buckner, 2014). Some of the main lines of research in this direction involved whole-brain network similarity analyses on the intrinsic and task-evoked network architecture of human connectome (Cole et al., 2014); the mapping of cortical hubs and brain region for adaptive task control (so-called cognitive control network (Cole et al., 2013; Cole & Schneider, 2007); and the investigation of activity flow from resting-state FCs to infer brain regions that carry diverse cognitive task information (Cole et al., 2013; Ito et al., 2017). Despite all these efforts in trying to characterize connectivity differences between resting-state and task activity in brain networks, a systematic analysis on how to measure pairwise (i.e., at the level of FC links) “cognitive distance” between these different functional states is still lacking. Such a methodology would provide a straightforward way to quantify differences in cognitive processing across tasks; also, it would help in relating these local differences in task-based FCs to the underlying structural network architecture, another exciting avenue for the brain connectomics community (Fukushima et al., 2018; Hermundstad et al., 2013; Hermundstad et al., 2014; Medaglia et al., 2018; Mišić et al., 2016; see also our recent work, Amico & Goñi, 2018).

Here we propose a framework, based on Jensen-Shannon (JS) divergence (Brit & Harremos, 2009; Cover & Thomas, 2012), to map the “cognitive distance” between task and resting-state functional connections. We show how this simple measure allows for quantifying the amount of changes in distributed and centralized processing in human functional networks.

We use resting-state and seven different task sessions from the Human Connectome Project (HCP) database to obtain the most JS-distant edges across tasks. We study how these changes across tasks are associated with different functional brain networks, and use the proposed measure to infer modifications in the information-processing regimes of these networks. Furthermore, we show how cognitive distance is shaped by the brain structural architecture and the level of nestedness of axonal pathways, and to what extent this relationship depends on the task. We conclude by discussing the new insights offered by this approach, as well as potential applications and future directions.

## METHODS

### Dataset

The fMRI dataset used in this work is from the publicly available Human Connectome Project (HCP, http://www.humanconnectome.org/), Release Q3. Per HCP protocol, all subjects gave written informed consent to the Human Connectome Project consortium. Below is the full description of the acquisition protocol and processing steps.

### HCP: Functional Data

We assessed the 100 unrelated subjects (54 females, 46 males, mean age = 29.1 years, *SD* = 3.7 years) as provided at the HCP 900 subjects data release (Van Essen, Smith, et al., 2013; Van Essen, Ugurbil, et al., 2012). This subset of subjects provided by HCP ensures that they are not family relatives. This criterion was crucial to exclude the need of family-structure covariables in our analyses as well as possible identifiability confounds. The fMRI resting-state runs were acquired in separate sessions on two different days (HCP filenames: rfMRI_REST1 and rfMRI_REST2), with two different acquisitions (left to right or LR, and right to left or RL) per day (Glasser et al., 2013; S. M. Smith et al., 2013). The seven fMRI tasks were gambling (tfMRI_GAMBLING), relational (tfMRI_RELATIONAL), social (tfMRI_SOCIAL), working memory (tfMRI_WM), motor (tfMRI_MOTOR), language (tfMRI_LANGUAGE, including both a story-listening and an arithmetic task), and emotion (tfMRI_EMOTION). The working memory, gambling, and motor tasks were acquired on the first day, and the other tasks were acquired on the second day (Barch et al., 2013; Van Essen, Smith, et al., 2013). The HCP scanning protocol was approved by the local Institutional Review Board at Washington University in St. Louis. For all sessions, data from both the left-right (LR) and right-left (RL) phase-encoding runs were averaged to calculate connectivity matrices. This operation was done for all seven fMRI tasks. Full details on the HCP dataset have been published previously (Glasser et al., 2013; S. M. Smith et al., 2013; Van Essen, Ugurbil, et al., 2012).

### HCP: Structural Data

We used DWI runs from the same 100 unrelated subjects of the HCP 900 subjects data release (Van Essen, Smith, et al., 2013; Van Essen, Ugurbil, et al., 2012). The diffusion acquisition protocol is covered in detail elsewhere (Glasser et al., 2013; Sotiropoulos et al., 2013; Uurbil et al., 2013). Below we mention the main characteristics. Very high-resolution acquisitions (1.25 mm isotropic) were obtained by using a StejskalTanner (monopolar) (Stejskal & Tanner, 1965) diffusion-encoding scheme. Sampling in q-space was performed by including three shells at *b* = 1,000, 2,000, and 3,000 s/mm^2^. For each shell corresponding to 90 diffusion gradient directions and five reference volumes (*b* = 0) were obtained, with the phase-encoding direction reversed for each pair (i.e., LR and RL pairs). Directions were optimized within and across shells (i.e., staggered) to maximize angular coverage using the approach of Caruyer et al. (2011; http://www-sop.inria.fr/members/Emmanuel.Caruyer/q-space-sampling.php), and form a total of 270 noncollinear directions for each phase-encoding direction. Correction for EPI and eddy current-induced distortions in the diffusion data was based on manipulation of the acquisitions so that a given distortion manifests itself differently in different images (Andersson, Skare, & Ashburner, 2003). To ensure better correspondence between the phase-encoding reversed pairs, the whole set of diffusion-weighted (DW) volumes is acquired in six separate series. These series were grouped into three pairs, and within each pair the two series contained the same DW directions but with reversed phase-encoding (i.e., a series of *M*_*i*_ DW volumes with RL phase-encoding is followed by a series of *M*_*i*_ volumes with LR phase-encoding, where *i* = [1, 2, 3]).

#### Brain atlas.

We employed a cortical parcellation into 360 brain regions as recently proposed by Glasser et al. (2016). For completeness, 14 subcortical regions were added, as provided by the HCP release (filename Atlas_ROI2.nii.gz). To do so, this file was converted from NIFTI to CIFTI format by using the HCP workbench software (Glasser et al., 2013; Marcus et al., 2011; http://www.humanconnectome.org/software/connectome-workbench.html, command -cifti- create-label).

### HCP Preprocessing: Functional Data

The HCP functional preprocessing pipeline (Glasser et al., 2013; S. M. Smith et al., 2013) was used for the employed dataset. This pipeline included artifact removal, motion correction, and registration to standard space. Full details on the pipeline can be found in Glasser et al. (2013) and S. M. Smith et al. (2013). The main steps were spatial (minimal) preprocessing, in both volumetric and grayordinate forms (i.e., where brain locations are stored as surface vertices; S. M. Smith et al., 2013); weak high-pass temporal filtering (>2,000 s full width at half maximum) applied to both forms, achieving slow drift removal; MELODIC ICA (Jenkinson, Beckmann, Behrens, Woolrich, & Smith, 2012) applied to volumetric data; and artifact components identified using FIX (Salimi-Khorshidi et al., 2014). Artifacts and motion-related time courses were regressed out (i.e., the six rigid-body parameter time series, their backwards-looking temporal derivatives, plus all 12 resulting regressors squared) of both volumetric and grayordinate data (S. M. Smith et al., 2013).

For the resting-state fMRI data, we also added the following steps: global gray matter signal was regressed out of the voxel time courses (Power et al., 2014); a bandpass first-order Butterworth filter in forward and reverse directions [0.001 Hz, 0.08 Hz] (Power et al., 2014) was applied (MATLAB functions *butter* and *filtfilt*); and the voxel time courses were z-scored and then averaged per brain region, excluding outlier time points outside of 3 standard deviation from the mean, using the workbench software (workbench command -cifti-parcellate). For task fMRI data, we applied the same above-mentioned steps but we opted for a more liberal bandpass filter [0.001 Hz, 0.25 Hz], since it is still unclear the connection between different tasks and optimal frequency ranges (Cole et al., 2014).

Pearson correlation coefficients between pairs of nodal time courses were calculated (MATLAB command *corr*), resulting in a symmetric connectivity matrix for each fMRI session of each subject. In this paper we will refer to this matrix as functional connectivity matrix or functional connectome (FC). As aforementioned, data from both the left-right (LR) and right-left (RL) phase-encoding runs were averaged to calculate individual functional connectomes in each fMRI session. Functional connectivity matrices were kept in its signed weighted form, hence neither thresholded nor binarized. Finally, the resulting individual functional connectivity matrices were ordered (rows and columns) according to seven functional cortical subnetworks (FNs) as proposed by Yeo and colleagues (Yeo et al., 2011). To do so, for each brain region in the Glasser atlas, the FN-membership (as a percentage) to each of the seven functional networks (sum of the membership vector being equal to 1) was calculated. Finally, each brain region was assigned to the most highly present FN. For completeness, an eighth subnetwork including the 14 HCP subcortical regions was added (as analogously done in recent papers; Amico & Goñi, 2018; Amico et al., 2017).

### HCP Preprocessing: Structural Data

The HCP DWI data were processed following the MRtrix3 (Tournier et al., 2011) guidelines (http://mrtrix.readthedocs.io/en/latest/tutorials/hcp_connectome.html). In summary, we first generated a tissue-segmented image appropriate for anatomically constrained tractography (ACT; R. E. Smith et al., 2012; MRtrix command 5ttgen); we then estimated the multi-shell, multi-tissue response function (Christiaens et al., 2015; MRtrix command dwi2response msmt_5tt) and performed the multi-shell, multi-tissue constrained spherical deconvolution (Jeurissen, Tournier, Dhollander, Connelly, & Sijbers, 2014; MRtrix dwi2fod msmt_csd). Afterwards, we generated the initial tractogram (MRtrix command tckgen, 10 million streamlines, maximum tract length = 250, FA cutoff = 0.06) and applied the successor of spherical-deconvolution informed filtering of tractograms (SIFT2; R. E. Smith et al., 2015) methodology (MRtrix command tcksift2). Both SIFT (R. E. Smith, Tournier, Calamante, & Connelly, 2013) and SIFT2 (R. E. Smith et al., 2015) methods provide more biologically meaningful estimates of structural connection density. SIFT2 allows for a more logically direct and computationally efficient solution to the streamlines connectivity quantification problem: By determining an appropriate cross-sectional area multiplier for each streamline rather than removing streamlines altogether, measures of fiber connectivity are obtained while making use of the complete streamlines reconstruction (R. E. Smith et al., 2015). Finally, we mapped the SIFT2 outputted streamlines onto the 374 chosen brain regions (360 from the Glasser et al., 2016, brain atlas plus 14 subcortical regions; see the Brain Atlas section) to produce a structural connectome (MRtrix command tck2connectome). Finally, a log_10_ transformation (Fornito et al., 2016) was applied on the structural connectomes (SC; i.e., the anatomical networks) to better account for differences at different magnitudes. In consequence, SC values ranged between 0 and 5 on this dataset.

### Jensen-Shannon Distance on Functional Edges

The Jensen-Shannon divergence is a method commonly used to measure dissimilarities between two probability distributions (Brit & Harremos, 2009; Cover & Thomas, 2012; De Domenico, Nicosia, Arenas, & Latora, 2015). In the case of two discrete probability distributions *P* and *Q*, the Jensen-Shannon divergence (*JSD*) is defined by

$$JSD(P||Q)=\frac{1}{2}D_{KL}(P||M)+\frac{1}{2}D_{KL}(Q||M),$$ (1)

where *M* = $\frac{1}{2}$(*P* + *Q*) and *D*_*KL*_ is the Kullback-Leibler divergence (Cover & Thomas, 2012). For two discrete probability distributions *P* and *Q*, it is defined by

$$D_{KL}(P||Q)=-\underset{i}{\sum }P(i)\log\frac{Q(i)}{P(i)}.$$ (2)

For the particular case of measuring the dissimilarity between two probability distributions *P* and *Q*, the Jensen-Shannon divergence is bounded between 0 and 1, given that one uses the base 2 logarithm:

0≤JSD(P||Q)≤1. (3)

It has been shown that the square root of the Jensen-Shannon divergence is a well-defined distance metric (Endres & Schindelin, 2003; Österreicher & Vajda, 2003), often referred to as “Jensen-Shannon distance”:

$$JS_{dist}(P||Q)=\sqrt{JSD(P||Q)}.$$ (4)

**Figure 1.**
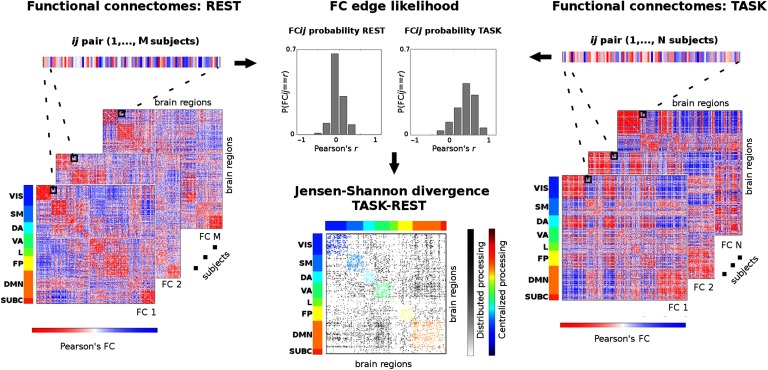
Workflow scheme for task-rest connectivity distance (unpaired). This scheme summarizes the procedure to measure edgewise distance from two cohorts of (M and N) functional connectomes (FCs) at rest (left) to a task-based one (right). First, an edge *ij* is extracted from the set, for both the resting-state and task-based FCs; these two vectors of M and N connectivity values are then transformed into probability distributions (center top); finally, the Jensen-Shannon distance for these two edgewise probabilities is computed (center bottom). Iterating this procedure over all possible *ij* pairs gives a Jensen-Shannon (JS) matrix of local distance in task FCs with respect to the REST baseline. The JS matrix is ordered by the seven functional networks (FNs): visual (VIS), somatomotor (SM), dorsal attention (DA), ventral attention (VA), limbic (L), frontoparietal (FP), and default mode network (DMN). An eighth subcortical network (SUBC) is added for completeness. Within-network most distant edges are color coded according to FNs. Between-network most distant edges are in gray scale. This method allows for quantifying the changes between centralized (within-network) and distributed (between-network) processing when a specific task is performed with respect to the resting-state baseline.

We used the *JS*_*dist*_ to map “connectivity distance” between resting-state and task sessions. Here we assume resting state to be the “cognitive baseline,” and we measured the *JS*_*dist*_ link to link from every task FCs to resting-state FCs. Below follows a detailed description of the procedure (see also the scheme at Figure 1). First, for every edge in a functional connectome, we extracted the corresponding individual values (out of 100 HCP subjects, we picked 50 subjects for resting-state FCs and 50 different subjects for the task FCs). In this study, this resulted in having two vectors with 50 elements, one for each resting-state edge and one for each edge in the task FCs whose JS distance is to be evaluated. These vectors represent Pearson’s correlation distributions of connectivity values across all subjects in the cohort. Secondly, we transform these two Pearson’s distributions into discrete probability distributions. We sampled the [−1, 1] Pearson’s range via uniform binning (bin width = 0.2), and counted the likelihood of occurrence of the connectivity values in each bin. Finally, the *JS*_*dist*_ between these two probability distributions was computed for every edge and HCP task considered in this study. This edgewise functional connectivity distance from resting state can be seen as a task-specific connectivity distance. That is, how far the distribution of values is in a specific task with respect to the resting-state FC baseline.

The Jensen-Shannon distance as defined above is thought for the general case where the two distributions *P* and *Q* come from different data samples (e.g., different subjects). This is not the most proper assessment in situations (like in the HCP data) where repeated measurements from same subjects are available, allowing for paired comparisons between FCs. To cover the necessity of that, we extended the concept of Jensen-Shannon distance by proposing a “paired” version of *JS*_*dist*_, namely $JS_{dist}^{paired}$, as follows. Similarly to *JS*_*dist*_, for every edge in a functional connectome, we extract the corresponding individual values. Then we take the element-wise difference between the two vectors, where subjects are indexed in the same order. Such a difference vector is then used to obtain distribution *P*([−2, 2] range, with uniform binning size of 0.1). We then compare *P* against a “null” distribution *Q*, which is probability 1 at the bin including the 0 value, and 0 for all other bins. Such *Q* distribution reflects the expectation of having no difference between repeated measurements. Deviations of *P* with respect to *Q* on functional edges reflect individual changes (in any direction and/or magnitude) between repeated measurements on subjects. Finally, we iterate this procedure for every edge and task to compute $JS_{dist}^{paired}$.

### Centralized and Distributed Processing in Functional Connectomes

**Figure 2.**
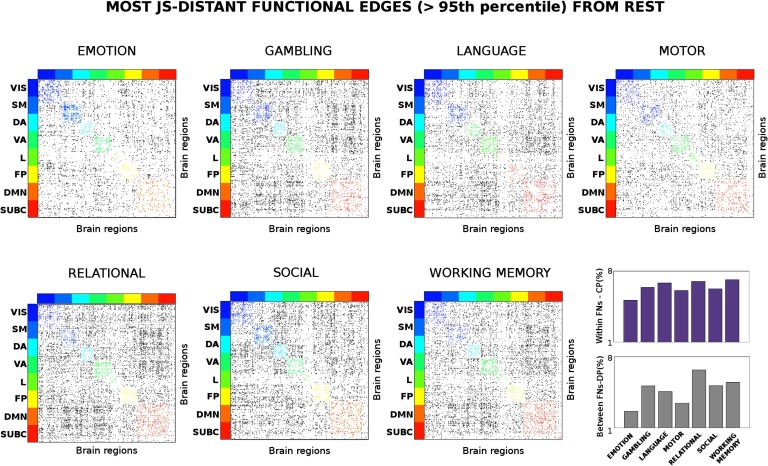
Connectivity distance across different tasks. Evaluation of the most distant functional links (in terms of Jensen-Shannon [JS] distance; see Methods) across seven different task sessions. The JS matrices were thresholded at the 95th percentile of the distribution of JS values across the seven tasks. The JS matrices were then ordered by seven functional networks (FNs; Yeo et al., 2011); visual (VIS), somatomotor (SM), dorsal attention (DA), ventral attention (VA), limbic (L), frontoparietal (FP), and default mode network (DMN). An eight subcortical network (SUBC) was added for completeness. The edges surviving the threshold corresponding to within-FN connections are color-coded accordingly. Edges corresponding to between-FN connections are depicted in gray scale. Note how the connectivity distance depends on the task: in some cases within-FN connectivity is more recruited (i.e., for the emotion task), in other between-FN connections are the most distant (i.e., relational task). The bottom-right bar plots depict the average percentage of within-FN most distant edges, i.e., centralized processing (CP, violet bars) and the average percentage of between-FN edges, that is, distributed processing (DP, gray bars) across the different tasks.

The aforementioned procedure produced 374 × 374 (i.e., number of regions in the employed brain atlas) *JS*_*dist*_ matrices per each task. Next, we sought to relate the proposed connectivity distance measure with changes in functional processing across functional networks (FNs). To do so, we first thresholded the *JS*_*dist*_ matrices based on the 95th percentile of the entire *JS*_*dist*_ distribution of values across all tasks, to select only the most distant links from resting state (see Figure 2). Next, we quantify the amount of change in each of the eight functional FNs (see the HCP Preprocessing: Functional Data section for details on the chosen FNs) by counting the number of edges that survived the threshold divided by the total number of edges present in each FN. We can then formalize changes in centralized processing (CP), for each functional network *k*, as

$$CP^{k}=\frac{\underset{i,j\in WN^{k}}{\sum }\hat{JS_{dist}(i,j)}}{\underset{i,j\in WN^{k}}{\sum }WN^{k}(i,j)},$$ (5)

where $\hat{JS_{dist}}$ is the binary version of the *JS*_*dist*_ matrix thresholded at the 95 percentile (1 for surviving edges, 0 elsewhere) for a specific task, and *WN*^*k*^ is a binary matrix of the same size as $\hat{JS_{dist}}$, with 1 if an edge falls within functional network *k*, and 0 elsewhere. Similarly, one can quantify changes in distributed processing (DP) as

$$DP^{kl}=\frac{\underset{i,j\in BN^{kl}}{\sum }\hat{JS_{dist}(i,j)}}{\underset{i,j\in BN^{kl}}{\sum }BN^{kl}(i,j)},$$ (6)

where now *BN*^*kl*^ is a binary matrix of the same size as $\hat{JS_{dist}}$, with 1 if an edge falls between functional networks *k*, *l*, and 0 elsewhere.

Hence, for each one of the eight functional networks considered here (see the HCP Preprocessing: Functional Data section for details), one can obtain one value of CP and seven values of DP (considering all pairwise FN interactions), for a specific task. These values provide an estimate of the density of the most connectivity distant functional links across, within, and between FN connectivity. That is, the amount of local changes in distributed and centralized processing in each FN from baseline, defined as resting-state functional connectivity.

### Bandpass Filter Evaluation on Centralized and Distributed Processing Analysis

In order to check whether the different bandpass ranges applied for resting state and task had an impact on centralized and distributed processing in FCs, we applied the same (liberal, i.e., [0.001*Hz*, 0.25*Hz*]) bandpass filter onto resting-state data. We then evaluated changes in centralized and distributed processing after the new bandpass, and checked the similarity with the “standard” bandpass results by computing the cosine similarity between the vectors defined by centralized and distributed processing coordinates. Cosine similarity is a measure of similarity between two nonzero vectors of an inner product space that measure the angle between them. Here, each vector represents a point in the state space defined by centralized and distributed processing.

### Null Model Evaluation for Connectivity Distance Analysis

To validate the *JS*_*dist*_ results in functional connectomes, we tested the same approach on randomized counterparts (or “surrogates”) of the original data. To do so, we employed the amplitude adjusted fourier transform (AAFT) surrogates method (Schreiber & Schmitz, 2000) to obtain data random surrogates. Starting from the 374 fMRI time series (one per brain region in the atlas; see also the Brain Atlas section), we generated AAFT fMRI time series surrogates as proposed in (Schreiber & Schmitz, 2000). This method aims to build surrogate time series that preserve the amplitude distribution and the power spectrum of the original data (Schreiber & Schmitz, 2000).

For each of the seven HCP tasks and resting state, we computed 100 surrogate versions of the functional connectivity matrices, and then evaluated the number of nonzero elements in ${\hat{JS_{dist}}}_{\text{surrogate}}$ for each of the 100 realizations. This provided null distributions (one per task) for the connectivity distance measure, which allowed us to test whether the results obtained on the original FCs were statistically significant.

Finally, in order to test the significance of differences between pairs of (highly structured) correlation matrices, we also employed a permutation test that preserved intact the correlation structure of the FC matrices but randomly permuted the task/rest labels, computed the JS distance on the permuted data, and then evaluated the number of JS edges per task surviving the 95% threshold based on the original data.

### Estimation of Functional Connectivity Distance Associations with Structural Connectomes

Next, we sought to assess the role of structural connections in the connectivity distance of functional links across all seven tasks. In order to do so, we divided the group-averaged structural connectivity (SC) weights (see the HCP Preprocessing: Structural Data section for details on SC computation) into five different percentile intervals {0–20; 20–40; 40–60; 60–80; 80–100}. We then counted the average number of most distant edges (i.e., the nonzero elements of $\hat{JS_{dist}}$) falling in each of the five percentile intervals, for each of the seven HCP tasks. This provides an estimate on the relationship between structural connections and connectivity distance and whether it depends on the specific task being performed. We also tested whether centralized and distributed processing depend on the “nestedness” or “hiddenness” of the structural pathways, as measured by search information (Goñi et al., 2014; Rosvall, Grönlund, Minnhagen, & Sneppen, 2005; Trusina, Rosvall, & Sneppen, 2005). Search information (SI) quantifies the accessibility or hiddenness of the shortest path between a source node and a target node within the network by measuring the amount of knowledge or information in bits needed to access the path (Goñi et al., 2014; Rosvall et al., 2005; Trusina et al., 2005; Wirsich et al., 2016). The more nested the shortest path is between two brain regions *ij*, the higher its SI value; conversely, the less hidden or integrated the path, the lower its SI value. Similarly to the experiment performed on SC weights, we again divided the group-average SI range of values into five different percentile intervals: {0–20; 20–40; 40–60; 60–80; 80–100}. Finally, we counted the average number of most distant edges (i.e., the nonzero elements of $\hat{JS_{dist}}$) falling in each of the five SI percentile intervals, for each of the seven HCP tasks. This provides an estimate on the relationship between structural “hiddenness” and connectivity distance and its associations with the specific task being performed. For both SC weights and SI, the significance of the associations with centralized and distributed processing was assessed through one-way analysis of variance (ANOVA; Hogg & Ledolter, 1987; MATLAB command *anova1*), with “observations” being centralized and distributed processing values for the seven tasks, and “groups” being the five percentile intervals described above.

## RESULTS

The dataset used for this study consisted of functional data from the 100 unrelated subjects in the Q3 release of the HCP (Van Essen, Smith, et al., 2013; Van Essen, Ugurbil, et al., 2012). We defined the “connectivity distance” between task FC links and resting-state FC links as the edgewise Jensen-Shannon distance (*JS*_*dist*_) between resting-state FCs and task FCs (see also the scheme at Figure 1). This metric quantifies the connectivity distance of a functional link recruited in a task with respect to its correspondent “usage” in resting state. For each of the seven HCP tasks (see Methods for details), we computed the corresponding *JS*_*dist*_ and $JS_{dist}^{paired}$ matrices, and extracted the most connectivity distant edges (≥ 95 percentile distribution of *JS*_*dist*_ and $JS_{dist}^{paired}$ values across all tasks). Figure 2 shows the results corresponding to *JS*_*dist*_ (unpaired, all different subjects for resting-state and task FCs), and Figure S1 (Amico, Arenas, & Goñi, 2019) summarizes the results for $JS_{dist}^{paired}$ (paired, same subjects for resting-state and task FCs). Please also see the Jensen-Shannon Distance on Functional Edges section for details.

Notably, the results obtained are significantly different from the same analyses performed on 100 realizations of surrogate data built from the fMRI time series considered in this study (Figure S2 and Table S1, Amico et al., 2019); see the Null Models Evaluation for Connectivity Distance Analysis section for details). Furthermore, with the only exception of motor versus emotion for absolute frame displacement, no significant differences were observed in frame-wise displacement estimates (*p* < 0.01, double-sided *t* test between task pairs). This included absolute frame displacement (root mean squared, HCP filename Abs_RMS) and relative frame displacement (root mean squared, HCP filename Rel_RMS). These findings suggest that head motion is not biasing rest to task JS distances depicted in Figure 2 and Figure S1 (Amico et al., 2019).

**Figure 3.**
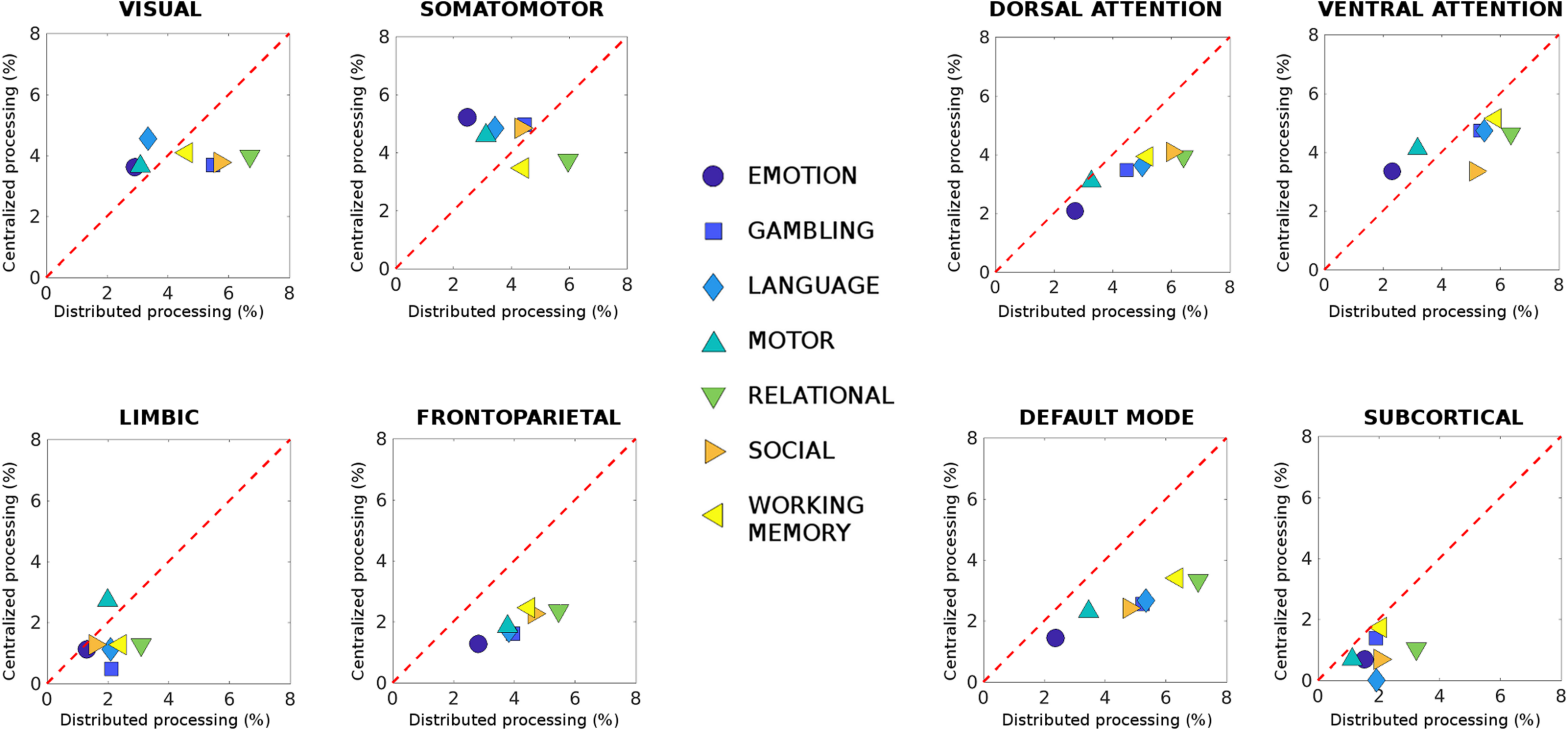
Centralized and distributed task processing in functional connectomes. Each plot shows differences in centralized versus distributed processing (see Methods) for each of the seven functional networks (FNs; visual, somatomotor, dorsal and ventral attention, limbic, frontoparietal, and DMN; Yeo et al., 2011) and subcortical network, for all seven different HCP tasks. The difference in centralized processing with respect to resting state was defined as the number of most Jensen-Shannon (JS) distant edges within-FN divided by the total number of edges in the FN (reported as percentage). Similarly, deviations from distributed processing in resting state were defined as the number of most JS-distant edges between FN divided by the total number of between-FN connections. Note how FP and DMN networks deviate from rest mainly in the amount of distributed processing, that is, between-FN connectivity.

Interestingly, the level of distance from resting state seems to be associated with the specific task (Figure 2). For some tasks, the within-functional network links are more distant, that is, more involved (e.g., for the emotion and motor tasks); in others the between-FN connections are the most distant ones (i.e., relational or working memory tasks). The dichotomy between intra-network (i.e., centralized) and inter-network (i.e., distributed) distance led us to quantify the changes in centralized and distributed processing in task FCs (Figure 3; also see the Centralized and Distributed Processing in Functional Connectomes section).

Note how, for three functional networks—dorsal, frontoparietal, and default mode—there is a clear demarcation between centralized and distributed processing, for all seven tasks evaluated (Figure 3). This indicates that the functional connections between these networks get more distant from rest when they are recruited in a task. Furthermore, with the exception of limbic and subcortical networks, where little difference in centralized and distributed processing can be observed (Figure 3), in all the other FNs (i.e., visual, somatomotor, and ventral attention) there is balance between intra- and inter-network processing. This trade-off seems to depend on the task at hand (slightly more centralized in some, more distributed in others; Figure 3). Note that these different distributions of centralized and distributed processing across tasks are not related to the different bandpass applied for rest and task data, since very similar results were obtained when the same (liberal, i.e., [0.001*Hz*, 0.25*Hz*]) bandpass parameters were applied onto resting-state data; (see Figure S3 in the supporting information, Amico et al., 2019).

**Figure 4.**
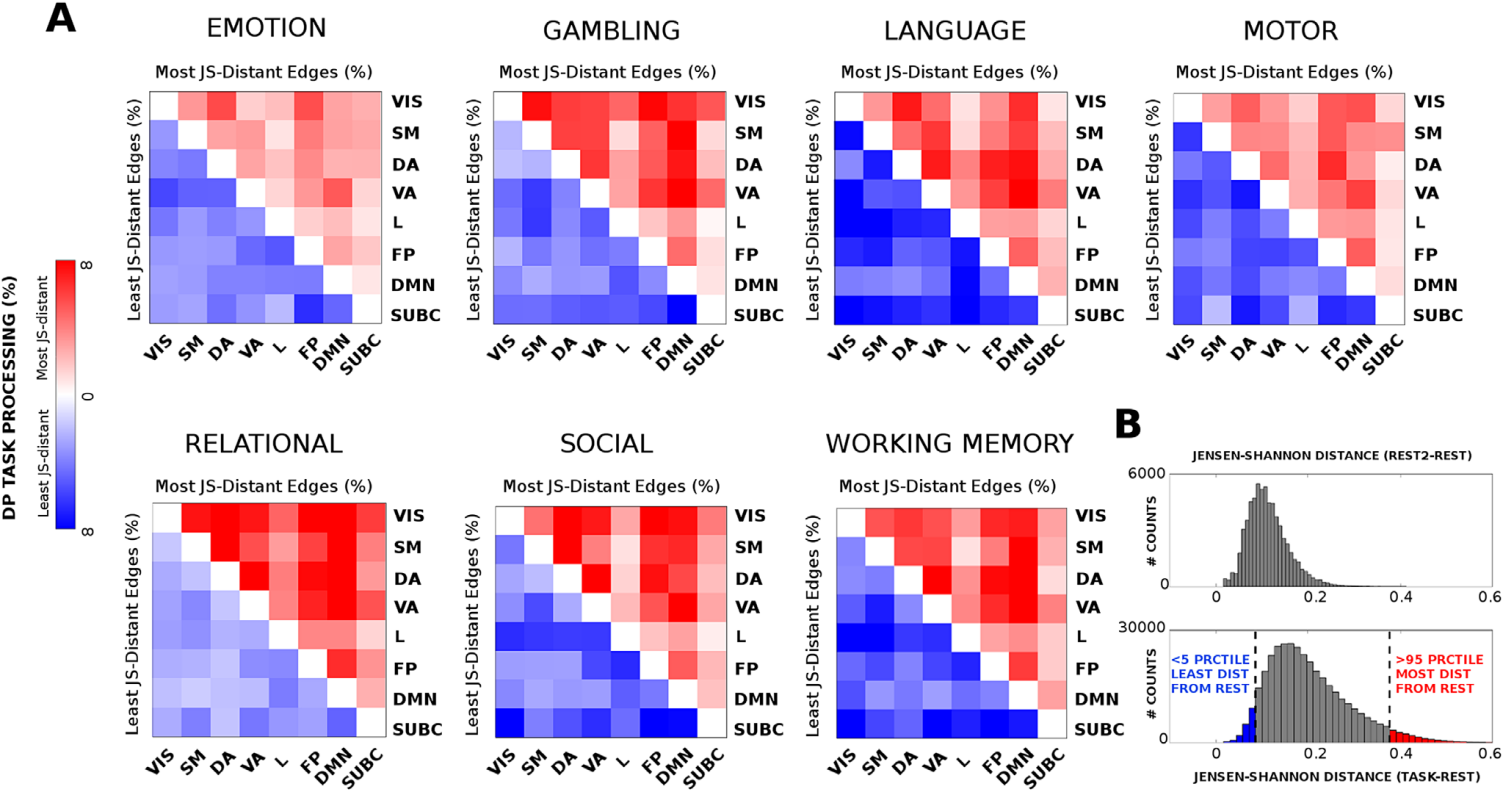
Least and most distant edges per functional network across tasks. (A) Heat maps, for all seven fMRI tasks evaluated, showing the most (red, upper triangular) and least (blue, lower triangular) distributed processing (DP) values between pairs of functional networks with respect to REST. (B) Top: distribution of JS-distance values when comparing REST2 session to the baseline REST session. Bottom: distribution of Jensen-Shannon distance values across the seven tasks evaluated. The tails of the histogram are highlighted in blue (least distant edges, < 5th percentile) and red (most distant edges, > 95th percentile).

When looking at JS-distance differences between functional networks across tasks, task-specific patterns may be observed (see Figure 4A). For instance, relational tasks exhibit a whole-brain tendency to get more distant from rest (i.e., higher general distributional processing); emotion or motor tasks are among the least distant from rest; whereas some other tasks (e.g., language or social) display a more specialized distributed processing across functional networks combining most and least distant functional edges. The histogram of the distribution of JS-distance values across all tasks gives more insights on the cognitive distance task-rest: there is a general tendency to be different from REST. However, some edges (<5%, Figure 4B, blue bars) stay almost unchanged with respect to REST configuration; others (>95%, Figure 4B, red bars) switch to more distant values, allowing for the cognitive reconfiguration of the system. Note that the JS distance does not depend on the baseline (i.e., REST FCs) magnitude of the correlations, as the average correlation between JS-values and median REST FC correlation values across tasks is −0.006 ± 0.026. Importantly, no single edge survived to the 95th percentile threshold after permutation testing of the TASK-REST labels (see Methods for details; Figure S4, Amico et al., 2019). Analogously, less than 1% of REST2-REST edges survived to that same threshold (Figure 4B). Overall, these analyses indicate that the conservative threshold chosen will depict actual task-rest FC changes.

**Figure 5.**
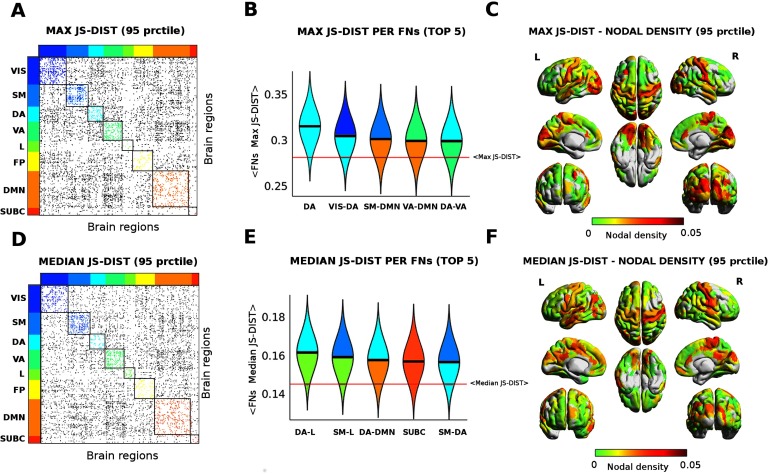
Functional reconfiguration via Jensen-Shannon distance. (A–D) Edgewise max (A) and median (D) Jensen-Shannon distance across all tasks (thresholded by 95th percentiles for max and for median). The colored dots depict JS values within FNs; gray dots indicate significant JS-distant edges between FNs. (B–E) Violin plot of edgewise JS distance (max and median) for the top five FNs and FN interactions. Within-FNs are color coded accordingly (as in A–D), while between-FNs are color coded using the colors of the two FNs involved. Solid black lines depict median values of each distribution; solid red lines indicate the whole-brain median value of max and median distributions. (C–F) Brain render of max and median JS distances as nodal density per region. The strength per brain region computed as sum of JS distance (max and median) for functional edges above the 95 percentile threshold divided by the total number of brain regions.

The results in Figure 4 display the extent to which JS distance is sensitive to different levels of functional reconfiguration (Krienen et al., 2014; Schultz & Cole, 2016; Shine et al., 2016) for different functional networks across different tasks. Hence we decided to explore further on this, and evaluated the edgewise maximum and median JS distance across all tasks. This would provide an overall summary of the main edges and functional networks involved in the cognitive switch across the seven tasks (see Figure 5). Notice that median and max give two different “flavors” of brain network reconfiguration: The median tells us how far, on average, brain regions (and functional networks) change when subjects perform tasks. The max tells us what are the most distant values, hence the highest achieved functional reconfiguration for a brain region across the seven different tasks under study.

**Figure 6.**
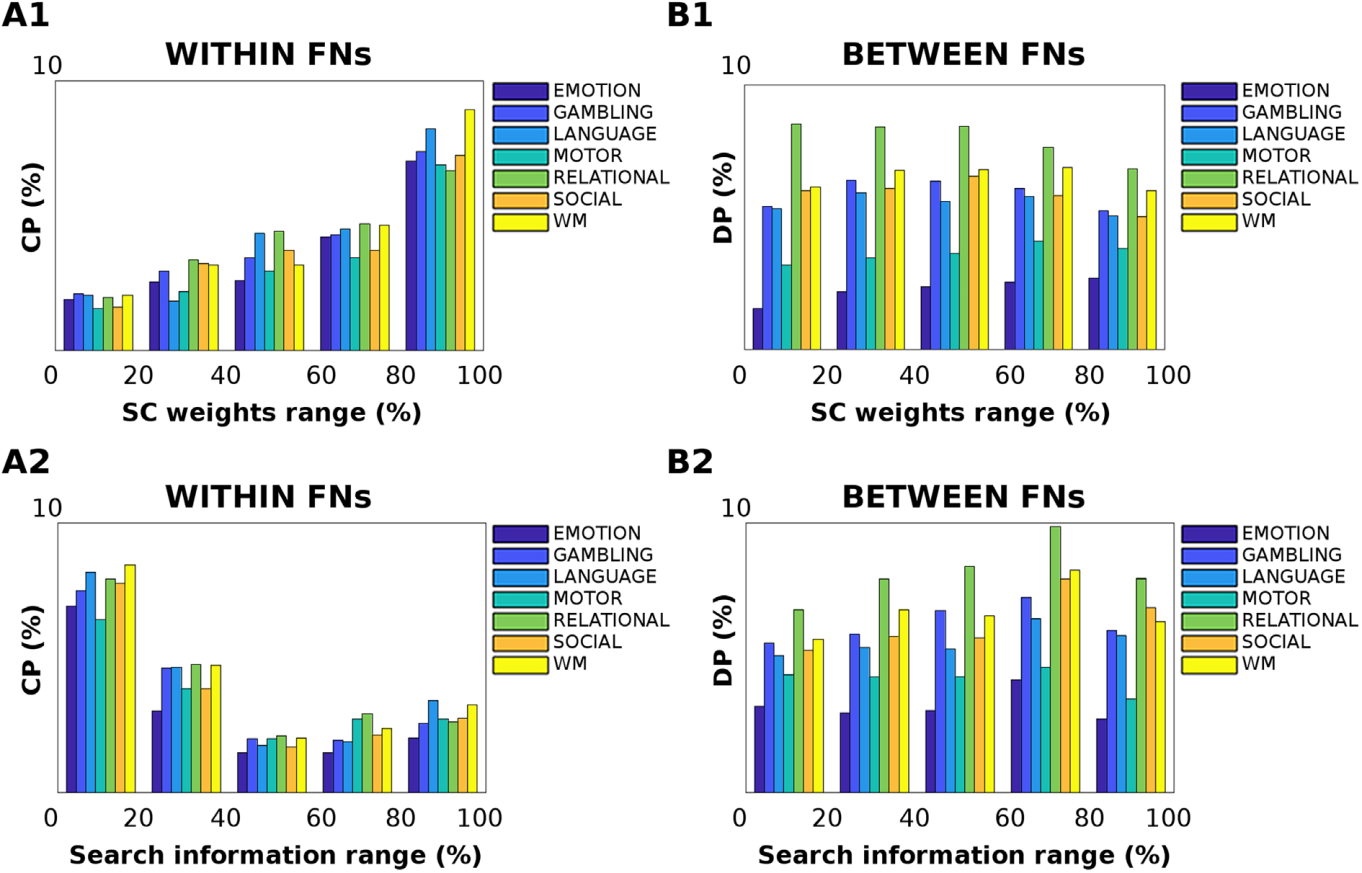
Effect of structural pathways on centralized and distributed processing changes. (A1–B1) The relationship between the anatomical connections and Jensen-Shannon distance was evaluated across the seven different tasks. The bar plots show the percentage of centralized processing (CP) within functional networks (FNs, A1) and distributed processing (DP) between FNs (B1), per five different percentile ranges of structural connectivity weights: 0–20, 20–40, 40–60, 60–80, and 80–100. The percentile range was extracted from the group-averaged structural connectome. Note how, for within-FN connections (A1), the change in centralized processing significantly correlates with the strength of structural connections across all tasks (one-way ANOVA *F* = 163.39, *df* = 4, *p* = 6.62 ⋅ 10^−20^); conversely, the underlying structural connectivity does not play a major role in distributed processing changes (one-way ANOVA *F* = 1.11, *df* = 4, *p* = 0.37). (A2–B2) The effect of structural path accessibility (as measured by search information; see Methods) on centralized and distributed processing was tested across the seven different tasks, per five different percentile intervals of search information: 0–20, 20–40, 40–60, 60–80, and 80–100. The percentile range was extracted from the group-averaged search information matrix. Notably, changes in centralized processing (A2) are significantly associated with low values of search information (one-way ANOVA *F* = 131.75, *df* = 4, *p* = 1.41 ⋅ 10^−18^); conversely, no significant association between SI and distributed processing changes was found (one-way ANOVA *F* = 1.85, *df* = 4, *p* = 0.14).

In order to determine whether changes in task processing are related to the underlying structural connectivity, we first evaluated the relationship between connectivity distance in each task and structural connectivity weights (Figure 6, A1–B1). Interestingly, a significant trend arises for all tasks between centralized processing and number of tracts (one-way ANOVA *F* = 163.39, *df* = 4, *p* = 6.62 ⋅ 10^−20^, Figure 6, A1). That is, the more structurally connected two regions are within a functional network, the higher the number of centralized processing edges recruited in a task. Notice how this trend is general and independent from the task, albeit the magnitude of this linear association between structure and function depends on the task at hand (Figure 6, A1). On the other hand, when looking at changes in distributed processing, that is, for edges involved in between-functional network connectivity, no significant associations with structural connectivity were observed (one-way ANOVA *F* = 1.11, *df* = 4, *p* = 0.37, Figure 6, B1).

We then dug deeper into the relationship between task processing and structural connectome by evaluating the level of hiddenness or accessibility of a structural path, as measured by search information (Goñi et al., 2014; see also the Estimation of Functional Connectivity Distance Associations with Structural Connectomes section for details), and testing its association with changes in cognitive task processing (Figure 6, A2–B2). Interestingly, the hiddenness of structural paths appears to be inversely related to changes in centralized processing (one-way ANOVA *F* = 131.75, *df* = 4, *p* = 1.41 ⋅ 10^−18^, Figure 6, A2). That is, the more “isolated” the structural pathway between two brain regions within a functional network, the higher will be its recruitment in a task. The more nested or integrated the path, the less distant the centralized processing with respect to resting state (Figure 6, A2). No significant associations were found when looking at changes in distributed processing versus search information range of values (one-way ANOVA *F* = 1.85, *df* = 4, *p* = 0.14, see Figure 6, B2).

## DISCUSSION

Cognitive brain network mapping (Cole et al., 2013; Cole & Schneider, 2007; Finn et al., 2017; Krienen et al., 2014), or the analysis of brain network features underlying task performance and cognitive control (Cole et al., 2014; Finn et al., 2017; Gratton et al., 2016; Khambhati, Medaglia, Karuza, Thompson-Schill, & Bassett, 2018; Tavor et al., 2016), is a recent and exciting new line of investigation in brain connectomics. While the general intrinsic common architecture between resting-state and task-based functional patterns has been explored (Cole et al., 2014), still very little is known about task connectivity distances and their associations with information processing (Cole, Ito, Bassett, & Schultz, 2016; Cole & Schneider, 2007; Ito et al., 2017). Furthermore, an even more intricate question relates to the relationship between the different task-based FC scenarios and the underlying structural connectivity (Amico & Goñi, 2018; Fukushima et al., 2018; Hermundstad et al., 2013; Hermundstad et al., 2014; Mišić et al., 2016).

Here we addressed these questions by proposing a novel methodology in neuroscience, rooted to the concept of Jensen-Shannon divergence (Brit & Harremos, 2009; De Domenico et al., 2015), to measure task-based pairwise functional distance with respect to the “cognitive baseline” defined by resting-state FCs (Figure 1). This framework may also be seen from a multilayer perspective, with the ground layer being resting-state FCs and top layers defined by the multiple task-based different connectivity scenarios. The distance defined here can be thought as interlayer coupling, or as the amount of cognitive processing necessary to make the “cognitive switch” from the resting-state ground layer to the top task-based functional layers. The JS divergence has several advantages: it is a nonparametric test, does not assume any form of distribution, and allows for quantifying fine-grained changes between two distributions. As shown in Figure S5 (Amico et al., 2019), there were a large number of functional edges for which REST or TASK FC distributions did not pass a normality test.

The work presented here complements the aforementioned recent studies on cognitive mapping, where the resting-state scaffolding was usually used to infer or also predict task changes in connectivity (Cole et al., 2014; Cole et al., 2016; Ito et al., 2017; Tavor et al., 2016). Here we evaluate and investigate the pairwise distance task-rest, and use it to map specific changes dictated by the task at hand. This adds up to previous studies in that it improves our understanding of how edge specific is the cognitive switch, and its level of recruitment (in terms of “connectivity distance”), as well as in terms of centralized and distributed processing changes in functional networks (Figure 2).

We exploited this new concept of connectivity distance to infer about the level of recruitment of an edge or of a functional network (Figure 2). Notably, the connectivity distant patterns present in the original data were significantly different from the ones obtained by surrogate data built from the original fMRI time series (Figure S2 and Table S1, Amico et al., 2019) or from the ones obtained by randomly shuffling rest-task FCs. Indeed, the more distant a functional network is in a specific task, the more different its recruitment with respect to resting state, and hence, the more changes in cognitive information processing that functional subsystem will undergo. This intuition led us to explore the concept of centralized and distributed processing in large-scale functional networks, which we defined as the difference between intra- (i.e., centralized) and inter- (i.e., distributed) network connectivity (Figure 3). Interestingly, three functional networks (dorsal attention, frontoparietal, and DMN) showed major changes in distributed processing and very minor changes in centralized processing, for all seven tasks evaluated with respect to resting state (Figure 3). This is in line with recent findings showing that frontoparietal and attentional areas appear to be the more flexible for cognitive control and task performance (Cole et al., 2013; Cole & Schneider, 2007; Krienen et al., 2014; Shine et al., 2016).

The fact that these networks and the DMN, which is well known to play a major role in resting state (Greicius, Krasnow, Reiss, & Menon, 2003; Raichle, 2015; Raichle et al., 2001), change mainly in terms of intercommunication when transitioning to task is also noteworthy. This finding goes along with the concept of integration of information between neural subsystems (Tononi, Sporns, & Edelman, 1994) and also with our recent findings on the association between FP-DMN disconnectivity and degradation in arousal and levels of consciousness (Amico et al., 2017). Possibly, the more demanding the task, the more the cross-talk between FP, DMN, attentional networks, and the rest of the brain might be needed to achieve the proper amount of cognitive processing or “brain network reconfiguration” (Krienen et al., 2014; Schultz & Cole, 2016).

We further investigated on the concept of brain network reconfiguration across tasks, by evaluating most and least distant functional edges between functional networks (Figure 4). Interestingly, some tasks seem to require extremely distant interactions between FNs (e.g., relational), and other tasks require specific subsets of FN interactions (e.g., language, working memory). The investigation of the max and median nodal *JS*_*dist*_ centrality (Figure 5) across tasks added more information on the complex scenario depicted in Figure 4. Dorsal and occipital regions seem to be the ones that are generally more distant from rest when engaged in a task (Figure 5, A–C). On the other hand, somatomotor and dorsal areas seem to be the ones that achieve the largest reconfiguration (i.e., maximal *JS*_*dist*_ from resting state) across the seven tasks evaluated (Figure 5, D–F).

Taken together, these findings suggest that the cognitive “switch” between resting-state and task states is more than a general shift in terms of functional links, but rather a complex interplay between maximally distant and minimally distant functional connections (Figure 4, Figure 5). This is in line with recent studies investigating the complex reconfiguration of brain networks during tasks (Bassett et al., 2013; Khambhati et al., 2018; Krienen et al., 2014; Shine et al., 2016).

Another major question relates to how these changes in cognitive processing are shaped or determined by the underlying structural architecture of a human brain. Very few studies so far have tried to elucidate the relationship between cognitive changes and axonal pathways, either for localized cortical subsystems (e.g., fusiform gyrus; Saygin et al., 2012) or for a specific task (e.g., visual stimuli; Osher et al., 2015), or at the whole-brain level (Hermundstad et al., 2013; Hermundstad et al., 2014; Mišić et al., 2016). In a recent work we tackled this problem from a whole-brain network perspective, by means of an ICA-based approach to extract the main “hybrid” functional-structural connectivity features sensitive to cognitive changes across seven different tasks (Amico & Goñi, 2018).

Here we took this investigation one step further by assessing functional connectivity distance associations with respect to the underlying structural connectivity weights (Figure 6). Interestingly, for changes in centralized processing, the relationship with structural connectivity is linear (Figure 6, A1). That is, when the cognitive processing involves mainly within-network connectivity, the higher the fiber strength is between two regions, the more distant they will be. Nonetheless, this relationship is not present when looking at distributed processing link-to-link effects (Figure 6, B1). Previous findings reported that between-network connectivity links can play a key role in the creation of more complex cognitive regimes (Fukushima et al., 2018; Hermundstad et al., 2013; Hermundstad et al., 2014; Mišić et al., 2016). The cross-talk between functional networks might bring the brain network up to a more integrated level, allowing for a more dynamic and distributed cognitive processing, that ultimately deviates far from the static underlying boundaries given by the structural fiber tracts.

To test this hypothesis, we evaluated the relationship between changes in distributed and centralized processing and structural path “hiddenness” or accessibility, as measured by search information (Goñi et al., 2014; see also the Estimation of Functional Connectivity Distance Associations with Structural Connectomes section). Notably, when looking at centralized processing deviations from rest, these two quantities appear to be inversely related (Figure 6, A2, B2). The less integrated the path is between two regions within a functional network, the higher the value in centralized processing, the more integrated the structural pathways, and the less centralized activity (Figure 6, A2).

These findings corroborate the hypothesis on the integration segregation in the human brain (Tononi et al., 1994). They are also in line with our findings on the importance of cross-talking between functional networks for task changes (Amico & Goñi, 2018), which can be summarized as follows: For a human brain to make a cognitive switch, a delicate interplay between centralized and distributed processing is necessary. The centralized activity within functional subsystems is shaped by brain structure. Moreover, the more isolated the shortest path connecting two centralized brain regions is, the higher the level of task processing (Figure 4). However, in order to achieve proper cognitive complexity for the task at hand, an appropriate level of distributed processing and subsequent integration between these subsystems is needed: The level of cross-talking and structural integration will depend on the specific task at hand (Figure 2, Figure 4) and on the functional subnetwork involved (Figure 3), with no significant function-structure associations (Figure 4).

This study has several limitations. The framework presented here is based on the quantification of distances between estimations of functional connectivity data. The accuracy and representativity of the estimate JS distance will be subject to the quantity and quality of the fMRI data as well as the processing steps. Further studies should explore how different aspects of the data and subsequent processing may have an impact in the JS-distance quantifications, including number of subjects, duration of the REST and TASK fMRI sessions, spatiotemporal limitations on fMRI data, and motion regressors included, among others. Analogously, our findings associating SC properties (weights and search information on SC shortest paths) with JS distance may also be, to some extent, sensitive to diffusion tractography user-defined free parameters. The effect of different brain atlases (here we used the one proposed by Glasser et al. (2016)) and functional network organization (here we used the one proposed by Yeo et al. (2011)) on the centralized and processing changes should be explored. Because of the way it is defined, the measure does not allow for individualized connectivity distance patterns. However, future studies could explore to what extent individual FCs can be predicted by group-wise changes in centralized and distributed processing, or use the information on the most distant link at the group level to select the most meaningful pairwise connectivity for the task at hand. Another potentially promising application of this methodology may be related to measuring JS divergence at the single-subject level with dynamic functional connectivity. In this case, the different FCs would be given by the FC snapshots obtained from sliding the FC window along the same subject acquisition during resting state and a specific task (as opposed to the slices being subjects as depicted in Figure 1 of this paper).

It is also important to remark on the importance of introducing a well-defined distance metric (i.e., *JS*_*dist*_) in the functional connectome domain, as we propose in this work. This can open several promising new lines of research in brain connectomics in terms of topological analysis of the connectivity domain, and of the definition of metrizable spaces where to map cognitive changes in brain functional networks. Finally, the simplicity of the methodology allows for its applicability in the clinical domain, where it could be employed to measure connectivity distance between “healthy” and “diseased” populations (e.g., Alzheimer’s, schizophrenia, coma), or to determine task-rest distance in situations where the cognitive switch is damaged or disrupted (e.g., in autism or similar neurological disorders).

In conclusion, we have reported a new methodology that aims at capturing the functional differences between different tasks when compared with resting state. The methodology based on the Jensen-Shannon distance is promising, and has been proved to discern between centralized and distributed activity across brain areas for different tasks. These results pave the way to the usage of this framework in other experiments, and to the development of a new information theoretical framework for the analysis of functional and structural connectomes.

## ACKNOWLEDGMENTS

Data were provided [in part] by the Human Connectome Project, WU-Minn Consortium (principal investigators: David Van Essen and Kamil Ugurbil; 1U54MH091657), funded by the 16 NIH Institutes and Centers that support the NIH Blueprint for Neuroscience Research; and by the McDonnell Center for Systems Neuroscience at Washington University.

## SUPPORTING INFORMATION

The code used for computing Jensen-Shannon distance in functional connectomes will be made available on the CONN*plexity* lab website (https://engineering.purdue.edu/ConnplexityLab).

## AUTHOR CONTRIBUTIONS

Enrico Amico: Conceptualization; Data curation; Formal analysis; Investigation; Writing – original draft; Writing – review & editing. Alex Arenas: Conceptualization; Methodology; Supervision; Writing – review & editing. Joaquín Goñi: Conceptualization; Investigation; Methodology; Project administration; Supervision; Validation; Writing – original draft; Writing – review & editing.

## FUNDING INFORMATION

Joaquín Goñi, National Institutes of Health (http://dx.doi.org/10.13039/100000002), Award ID: R01EB022574. Joaquín Goñi, National Institutes of Health (http://dx.doi.org/10.13039/100000002), Award ID: R01MH108467. Joaquín Goñi, Indiana Clinical and Translational Sciences Institute (http://dx.doi.org/10.13039/100006975), Award ID: UL1TR001108. Alex Arenas, Ministerio de Economía y Competitividad (http://dx.doi.org/10.13039/501100003329), Award ID: FIS2015-71582-C2-1. Alex Arenas, Institució Catalana de Recerca i Estudis Avançats (http://dx.doi.org/10.13039/501100003741). Alex Arenas, James S. McDonnell Foundation, (http://dx.doi.org/10.13039/100000913), Award ID: 220020325.

## TECHNICAL TERMS

Network: An ordered pair formed by a set of nodes and a set of edges (which represent connections between pairs of nodes). Nodes are usually represented by circles, whereas edges are represented by lines or arcs connecting pairs of nodes.
Diffusion-weighted imaging (DWI): A form of magnetic resonance imaging (MRI) technique based on measuring the random Brownian motion of water molecules within a voxel based on sampling 3D directions. This technique allows for estimating white matter streamlines and fiber bundles connecting brain regions.
Tractography: Computational reconstruction procedure that may be used to obtain, from DWI data, white matter streamlines or fiber tracts connecting different brain regions.
Functional magnetic resonance imaging (fMRI): A noninvasive technique that estimates brain activity by detecting changes associated with blood flow. The rationale of this technique relies on the fact that there is a positive association between cerebral blood flow and neuronal activation.
Connectome or structural connectivity (SC) matrix: A network representation of the physical connections in the brain. Nodes represent brain regions, whereas edges represent physical connections of pairs of brain regions through the white matter. Weights typically denote the density or the integrity of the connections.
Jensen-Shannon divergence: In probability theory and statistics, the Jensen-Shannon divergence is a method of measuring the dissimilarity between two probability distributions. The square root of the Jensen-Shannon divergence is a metric often referred to as Jensen-Shannon distance.
Functional connectome/connectivity (FC) matrix: A network representation of the functional coupling between brain regions. Such coupling is usually measured by quantifying the statistical dependencies between time series of brain regions (e.g., pairwise Pearson’s correlation, mutual information) as obtained by functional magnetic resonance imaging (fMRI).
Search information: A measurement that quantifies the accessibility or hiddenness of the shortest path between a source node and a target node within the network by measuring the amount of knowledge or information (expressed in bits) needed to access that exact path.
Metric: In mathematics, a metric or distance function is a function that defines a distance between each pair of elements of a set and satisfies the properties of nonnegativity, identity of indiscernibles, symmetry, and triangle inequality. A set with a metric is called a metric space.
