# Supplementary material for: Centralized and distributed cognitive task processing in the human connectome
Source: Netw Neurosci. 2019 Feb 1;3(2):455–74. doi: 10.1162/netn_a_00072 (PMC6370483; doi:10.1162/netn_a_00072)
Supplement: Supplementary file 1 [file netn-03-455-s001.pdf]

Amico, E., Arenas, A., & Goni, J. (2019). Supporting Information for "Centralized and Distributed Cognitive Task Processing in the Human Connectome." *Network Neuroscience*, *3*(2), 455–474. https://doi.org/10.1162/netn_a_00072

SUPPORTIVE INFORMATION

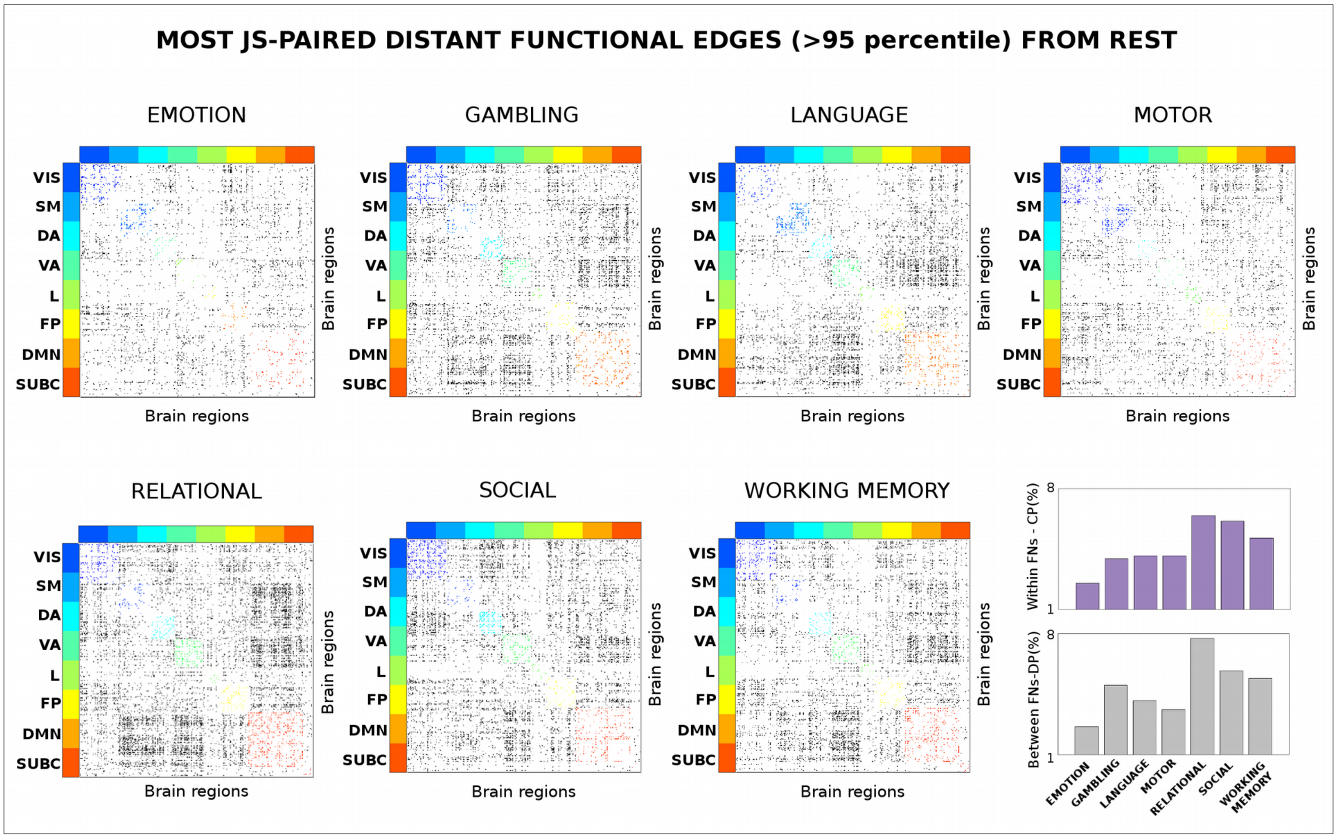

Figure S1. Connectivity distance across different tasks ("paired" Jensen-Shannon distance). Evaluation of the most distant functional links (in terms of Jensen-Shannon (JS) distance, "paired" version, see Methods) across 7 different task sessions. The JS matrices were thresholded at the 95% of the distribution of JS values across the seven tasks. The JS matrices then ordered by 7 functional networks (FNs, (Yeo et al., 2011)): visual (VIS), somato-motor (SM), dorsal attention (DA), ventral attention (VA), limbic (L), frontoparietal (FP), default mode network (DMN). An eight subcortical network (SUBC) was added for completeness. The edges surviving the threshold corresponding to within-FN connections color-coded accordingly. Edges corresponding to between-FN connections are depicted in gray-scale.

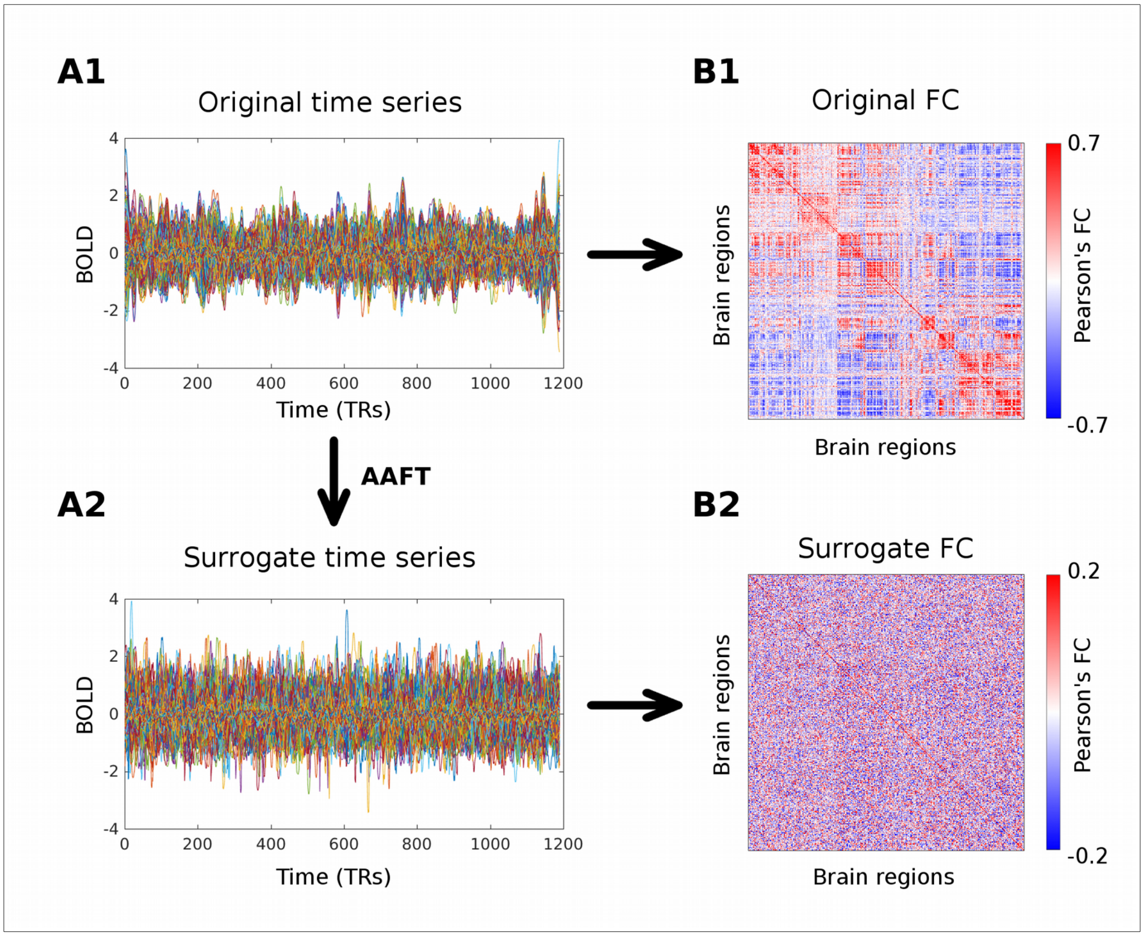

Figure S2. Scheme of the functional connectome randomization procedure. In order to validate the JSD results in functional connectomes (FC), we used "surrogates" of the original data. We here show an example of this procedure for one subject, resting-state. The original BOLD 374 (i.e. one per brain region) time series (A1), from which the FC was originated (B1), were randomized by means of the Amplitude Adjusted Fourier Transform (AAFT) surrogates method (Schreiber & Schmitz, 2000), see section *Null model evaluation for connectivity distance analysis* for details). These randomized time-series (A2) were then used for the construction of the surrogate FC of the subject (B2).

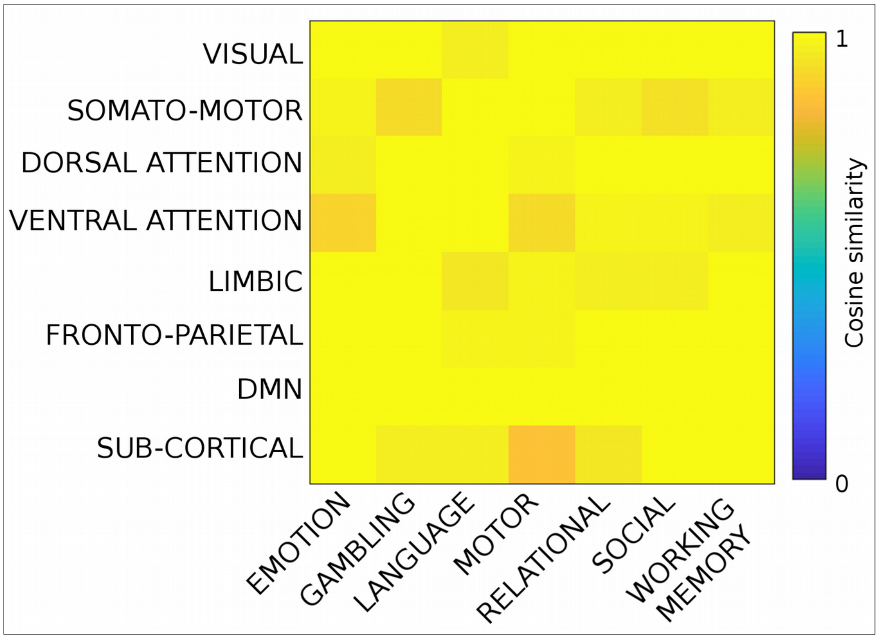

Figure S3. Evaluation of the effect of the bandpass filter for REST FCs. Figure shows the cosine similarity (see Methods) between pairs of {CP,DP} processing values (see Figure 3) for the two different frequency bands assessed ([0.001Hz to 0.08Hz] and [0.001Hz to 0.25Hz]).

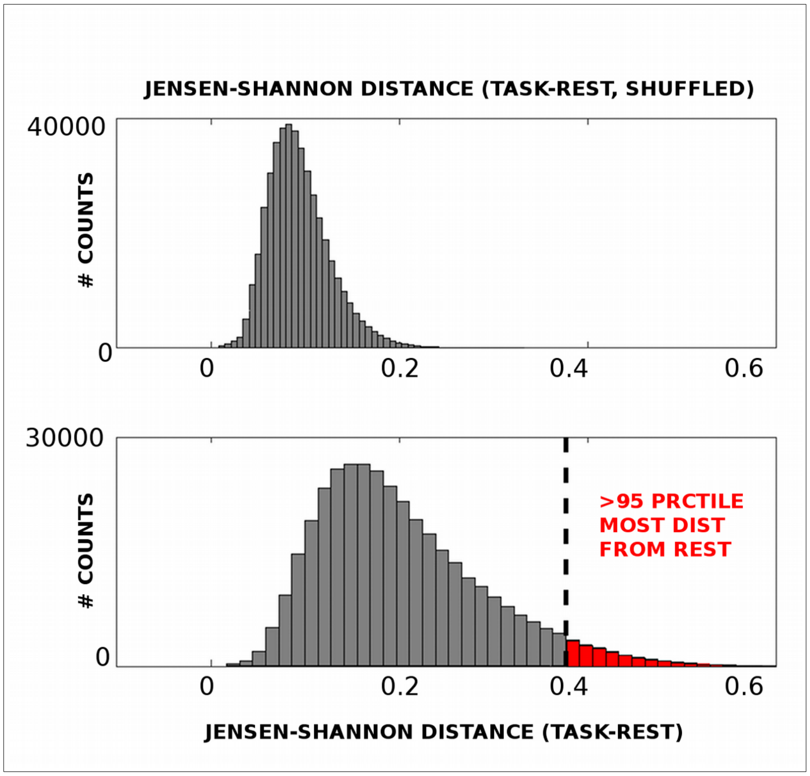

Figure S4: Null model for cognitive distance: TASK-REST label permutation test. Top histogram: distribution of JS distance values when randomly permuting the TASK/REST labels (see Null model evaluation for connectivity distance analysis for details). Bottom histogram: actual distribution of Jensen-Shannon distance values across the seven tasks evaluated. The right tail of the histogram is highlighted in red (most distant edges, > 95th percentile), corresponding to the chosen cutoff for centralized and distributed processing evaluation.

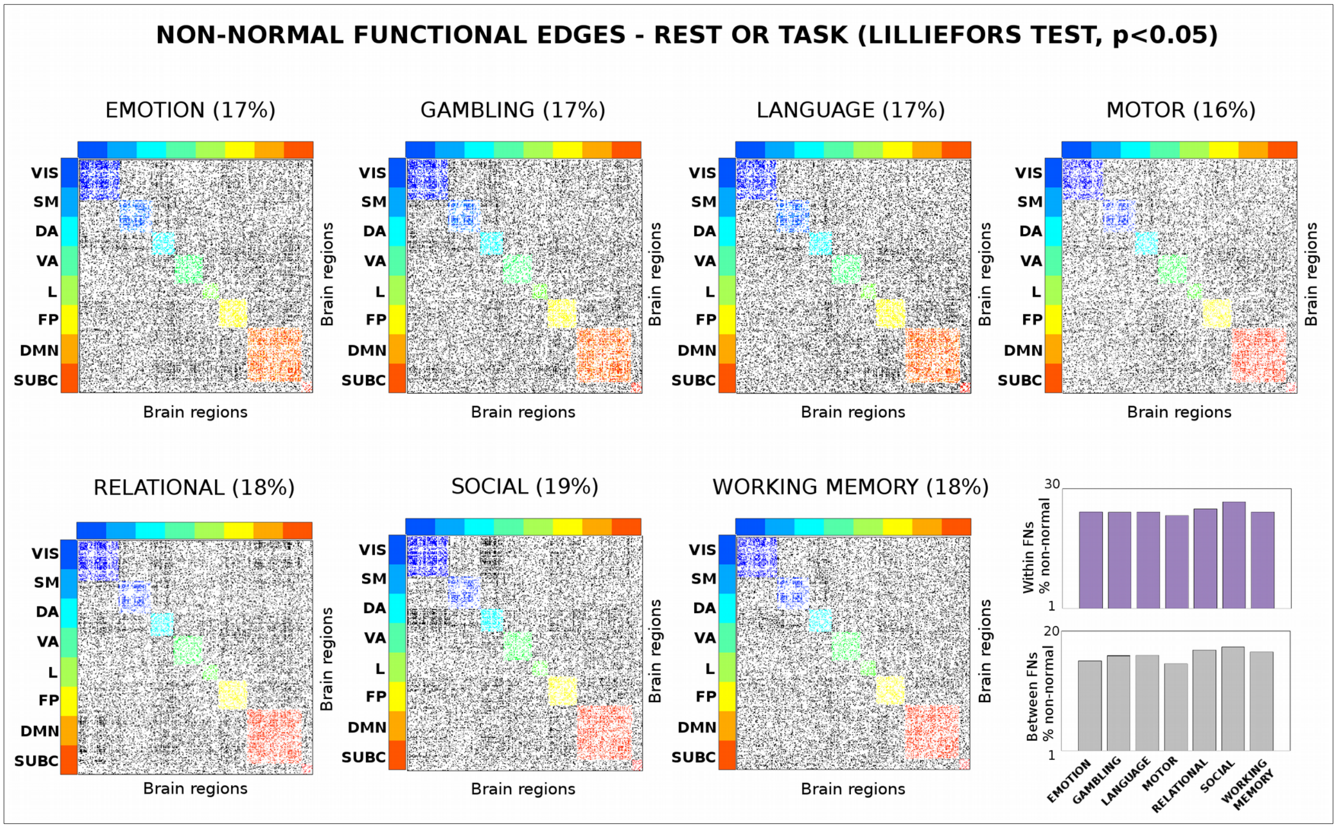

Figure S5. Normality test over functional edges. Results for Lilliefors test, edgewise for each task. Edges that did not pass the normality test (either at rest or on the assessed task) corresponding to within-FN are color-coded accordingly. Edges that did not pass the normality test corresponding to between-FN are depicted in grayscale. The percentage of non-normal edges per task is reported on top of each matrix. The bottom-right bar plots depict the average percentage of within-FNs non-normal edges (violet bars) and the average percentage of between-FNs non-normal edges (grey bars) for each task.

Task name	Most divergent edges Surrogate - median ± 99%	Most divergent edges Original values
Emotion	132 ± 36	1655
Gambling	18 ± 14	3736
Language	8 ± 9	3648
Motor	4 ± 7	2625
Relational	40 ± 19	4856
Social	376 ± 46	3732
Working Memory	1 ± 3	4161

Table S1. Null models for cognitive distance. Table reports, for each task, the median (and ± 99% confidence intervals of the distribution) values for the most divergent edges obtained from 100 realization of FC surrogates. The surrogates were built from the original fMRI time series using the Amplitude Adjusted Fourier Transform randomization procedure ((Schreiber & Schmitz, 2000), see *Null model evaluation for connectivity distance analysis* for details. Note how the original values of most distant edges are always significantly different from the surrogate null distribution (i.e. above the 99 percentile), for all the seven HCP tasks considered in this study.
